# Investigation et riposte à une épidémie de poliovirus sauvage à Kinshasa

**DOI:** 10.11604/pamj.2013.15.37.2665

**Published:** 2013-05-31

**Authors:** Muriel Nzazi Nsambu, Léodegal Bazira, Tiekoura Coulibaly, Albert Mbule, Michèle Dramaix Wilmet, Joris Losimba Likwela

**Affiliations:** 1 Médecin Epidémiologiste Coordonateur, Organisation Mondiale de la Santé, Sous-Bureau de Kinshasa, Avenue des Cliniques, Gombe, Kinshasa; 2 Représentant, Organisation Mondiale de la Santé, République Démocratique du Congo, Avenue des Cliniques, Gombe, Kinshasa; 3 Focal Point of Immunization Vaccine and Development, Organisation Mondiale de la Santé, République Démocratique du Congo, Avenue des Cliniques, Gombe, Kinshasa; 4 Organisation Mondiale de la Santé, République Démocratique du Congo, Avenue des Cliniques, Gombe, Kinshasa; 5 Centre de Recherche Epidémiologie, Biostatistiques et recherche clinique, Ecole de Santé Publique, Université Libre de Bruxelles, Belgique, Campus Erasme - CP598, 808 route de Lennik, 1070 Bruxelles; 6 Département de Santé Publique, Faculté de Médecine, Université de Kisangani, BP 2012, Kisangani, République Démocratique du Congo

**Keywords:** Paralysie flasque aigüe, poliovirus sauvage, épidémie, vaccination, République Démocratique du Congo, Acute flaccid paralysis, Wild Polio Virus, outbreak, vaccination, Democratic Republic of Congo

## Abstract

**Introduction:**

La République Démocratique du Congo a été considérée comme un pays à circulation rétablie de poliovirus sauvage (PVS). Cet article décrit l’épidémie de PVS qui a sévit dans la province de Kinshasa de 2010 à 2011.

**Méthodes:**

Les analyses ont porté sur les cas de paralysie flasque aigüe (PFA) enregistrés de décembre 2010 à décembre 2011, les données de surveillance des PFA, les données de couverture vaccinale et celles du monitorage indépendant des activités de vaccination supplémentaires.

**Résultats:**

Entre décembre 2010 à décembre 2011, 298 cas de PFA ont été enregistrés par les zones de santé parmi lesquels 34 cas de PVS confirmés. 58% des cas de PVS avaient plus de 15 ans avec plus d'hommes que de femmes. 10 passages d'activités de vaccination supplémentaires ont été mis en œuvre dont 4 avaient ciblé toute la population de Kinshasa. Il n'y a plus eu de cas de PVS après le 3e passage. Le monitorage des activités de vaccination a montré une proportion de sujets non vaccinés allant de 4 à 13%. La performance du système de surveillance était globalement bonne.

**Conclusion:**

La prédominance des adultes parmi les cas notifiés traduit leur susceptibilité alors qu'ils ne sont généralement pas concernés lors des campagnes de vaccination supplémentaires. Ceci devrait engager les autorités sanitaires à envisager des activités vaccinales supplémentaires ciblant les adultes afin de casser plus rapidement la chaîne de transmission. Les faiblesses subsistant dans le système de surveillance pourraient être jugulées par le renforcement de la surveillance à base communautaire.

## Introduction

La poliomyélite est une maladie infectieuse, invalidante et parfois mortelle qui affecte généralement les individus à l′aube de la vie [1]. Elle est causée par le poliovirus de type 1, 2 ou 3 [2]. Le poliovirus sauvage, ne disposant que de l′homme comme réservoir, est un virus ciblé pour l′éradication à l′instar du virus de la variole [3]. L′initiative pour l’éradication de la poliomyélite lancée par l′OMS en 1988 lors de la 41^e^ assemblée mondiale a permis une réduction de 99% de cas de polio dans le monde entre 1988 et 2000. Entre 1988 et 2005, le nombre de pays endémiques est passé de 125 à 4 [1, 3]. Le type 2 n′a plus été isolé dans le monde depuis 1999 [1, 2].

En 2012, cependant, il reste encore en Afrique un seul pays endémique, le Nigéria, et trois autres classés « pays à circulation rétablie »: l'Angola, le Tchad et la République Démocratique du Congo (RDC). Certains autres pays avaient eu des épidémies de poliomyélite en 2011 suite à l'importation du poliovirus principalement depuis le Nigeria. La plupart de ces pays appartiennent à la ceinture de l'importation du poliovirus sauvage, « wild poliovirus importation belt », s’étendant de l'Afrique de l'ouest, l'Afrique centrale jusqu’à la corne de l'Afrique [1]. Le Congo-Brazzaville, voisine de la RDC, a eu une épidémie de poliomyélite en 2010-2011 où les jeunes adultes ont été les plus touchés [4].

De 2000 à 20055, la RDC n'avait rapporté aucun cas de poliovirus sauvage. L'importation de poliovirus sauvage en RDC a débuté en 2006 où 13 cas avaient été notifiés liés à une souche importée de l′Angola [5]. Entre 2007 et 2009, 49 cas ont été rapportés. Les années 2010 et 2011 ont connu des flambées avec 1100 cas et 93 cas rapportés respectivement. La province de Kinshasa a été la plus touchée en 2011 [1].

Kidd S et collaborateurs avaient montré le lien entre la présence dans le ménage d'un adulte ayant voyagé en dehors de la province et le risque de paralysie flasque aigue (PFA) à poliovirus sauvage (PVS) [6]. La situation de la ville de Kinshasa, voisine de l′Angola et du Congo-Brazzaville (géographiquement et économiquement) et point de convergence des provinces du pays dont certaines ont été frappées d’épidémie en 2010 (Bandundu et Kasaï occidental), peut avoir contribué à l’éclosion de l’épidémie de 2011 [4, 6–7].

Le présent article décrit cette flambée et les activités de riposte organisées pour faire face à cette épidémie ainsi que des données de surveillance de la paralysie flasque aiguë (PFA) à Kinshasa.

## Méthodes

Les données relatives à l′investigation et la riposte à l′épidémie de poliomyélite qui a sévi à Kinshasa de décembre 2010 à juin 2011 et les données de surveillance sur une année (décembre 2010 à novembre 2011 de manière à couvrir toutes les activités de riposte organisées) ont été analysée rétrospectivement.

Sur le plan sanitaire, la RDC est découpée en provinces et ces dernières en districts sanitaires qui comprennent un nombre variable de zones de santé. Kinshasa, l'une des 11 provinces du pays, comprends 6 districts sanitaires et 35 zones de santé (ZS) : Gombe (4 ZS), Funa (4ZS), Lukunga (9ZS), Kalamu (8 ZS), Ndjili (7ZS) et Nsele (3 ZS).

Dans le cadre de la surveillance de routine, les cas de PFA sont notifiés par les relais communautaires dans le cadre de la surveillance à base communautaire ou par les points focaux de surveillance au sein des structures sanitaires ainsi que par les tradi-praticiens et les églises appelée « maison de guérison ». La recherche active est dévolue aux cadres des zones de santé, des districts sanitaires et de la Division Provinciale de la Santé (4e bureau chargé de la lutte contre la maladie et coordination provinciale du PEV). Ils assurent également le monitoring de la surveillance de routine. Les partenaires (OMS et UNICEF) viennent en appui à l'ensemble du processus et prennent part également à la surveillance active des PFA pour détecter des cas omis lors de la surveillance de routine.

La PFA est une maladie à notification immédiate. Tous les cas détectés font l'objet d'une investigation par les équipes des zones de santé, et sont par la suite validés par l’équipe de la Division Provinciale de la Santé et l'OMS. L'investigation est faite au moyen d'une d′une fiche d′investigation permettant de recueillir les informations relatives à l′identification du sujet, les données sociodémographiques, les manifestations cliniques, l′historique de la maladie (la date de début de la paralysie, la présence de la fièvre, la progression de la paralysie dans les 4 jours, l'asymétrie de la paralysie), le statut vaccinal et le moment de prélèvement d’échantillons, le suivi après 60 jours pour les cas inadéquats (cas prélevés après 14 jours suivant le début de la paralysie, cas avec intervalle de prélèvement des deux échantillons > 48h, quantité insuffisantes, échantillon arrivé en mauvaise condition au laboratoire).

Pour tous les cas de PFA notifiés, un prélèvement de 2 échantillons à un intervalle de 24 à 48 heures est réalisé et envoyé au laboratoire national de recherche biologique (INRB) dans une boîte isotherme contenant des accumulateurs de froid pour analyse, l'isolement du virus et le séquençage. Idéalement, les échantillons doivent être prélevés dans les 14 jours suivant le début de la paralysie.

Les données recueillies sur les formulaires d'investigation sont encodés dans une base des données sur Excel. Les analyses ont porté sur les cas de PFA et les cas de poliovirus sauvage (PVS) confirmé qui ont été notifiés depuis décembre 2010 jusqu'au mois de juin 2011. Le profil sociodémographique, vaccinal et clinique des cas de PFA a été décrit et le lien avec le PVS recherché à l'aide du test du chi carré au seuil de signification de 5%. Lorsque les conditions d'application du test de chi carré n’étaient pas satisfaites, le test exact de Fisher était utilisé.

La performance du système de surveillance des PFA a été évaluée à l'aide d'une série d'indicateurs standards retenu par l'OMS comme indicateur de performance de la surveillance des PFA, notamment:

- le taux annualisé de PFA non poliomyélitique dans la population des enfants de moins de 15 ans (normes OMS/AFRO : 2 cas pour 100 000 habitant);
- le pourcentage des selles adéquates c′est-à-dire prélevés dans les 14 jours suivant le début de la paralysie (norme = 80%), proportion de cas avec 2 échantillons prélevés dans un intervalle de 24 à 48 heures et pourcentage d’échantillons arrivant au laboratoire dans de bonnes conditions (bonne température, pas de suintement, fiches correctement remplies);
- la proportion de 2 échantillons de selles prélevés dans les 14 jours suivant le début de la paralysie;
- la proportion de cas de PFA investigués dans les 48h suivant la notification;

Afin de contrôler cette épidémie, il avait été organisé une série des campagnes de vaccination de toute la population en 4 passages à 1 mois d′intervalle et 4 passages chez les moins de 5 ans dans toutes les zones de santé. Les couvertures réalisées à chaque passage ont été calculées sur base des données recueillies sur les fiches de pointage par les vaccinateurs et centralisées par les zones de santé et rapporté sur la population totale estimée en fonction des projections faites partant du dernier recensement des populations en RDC.

Pour contribuer à l'amélioration de la qualité des campagnes mises en œuvre, une évaluation indépendante (monitorage indépendant) est réalisée pour chaque passage par des personnes extérieures au système de santé. Les données ayant fait l'objet des analyses sont:

- la proportion d'aires de vaccination ayant fait l'objet du monitorage,
- le nombre de personnes cible vus par les moniteurs,
- la proportion de personnes cible non vaccinées parmi les personnes cible vus dans les ménages,
- la proportion de personnes cible non vaccinés parmi les personnes cible vus hors ménages.

Les données ont été encodées et traitées à l'aide du tableur Excel et les analyses réalisées avec le logiciel STATA.11 for Windows.

## Résultats

De décembre 2010 à décembre 2011, 298 cas de PFA ont été notifiés et 34 cas de PVS de décembre 2010 à mai 2011. Le Tableau 1 présente la répartition des cas de PFA selon les groupes d’âge, le sexe, le district sanitaire et les principales manifestations cliniques observées. Il y avait près de la moitié des cas de PFA (47%) et près de 60% de PVS parmi les sujets âgés de 15 ans et plus. Outres les sujets de moins de 5 ans (20,6%), les tranches d’âges les plus affectées par le PVS étaient comprises entre 10 et 29 ans (70,7%). Le sexe ratio était de 1,7 en défaveur du sexe masculin. Il y avait significativement plus de cas de PVS dans le district sanitaire de Ndjili et de Lukunga.

**Tableau 1:** Répartition des cas en fonction de l’âge, du sexe, des districts sanitaires et des manifestations cliniques chez les sujets atteints de PFA (n = 298) et les cas d'infection à PVS (n = 34)

Caractéristique	PFA	PVS	Non PVS	P-val	Manifestations cliniques	PFA	PVS	Non PVS	P-val
	%	%	%			%	%	%	
n	298	34	264		n	298	34	264	
**Age**				0,33*	**Fièvre**				0.07£
< 5 ans	28,5	23,5	29,1		Oui	87,6	97,1	86,3	
5-14 ans	24,5	17,6	25,4		Non	10,1	0,0	11,4	
≥ 15 ans	47,0	58,8	45,5		Inconnu	2,3	2,9	2,3	
**Sexe**					**Paralysie flasque aigue**				0.66£
Féminin	36,9	29,4	37,9	0,34*	Oui	91,3	94,2	90,9	
Masculin	63,1	70,6	62,1		Non	6,4	2,9	6,8	
**District Sanitaire**				<0,01£	Inconnu	2,3	2,9	2,3	
Funa	5,7	0,0	6,4		**Paralysie asymétrique**				0.79£
Gombe	4,7	0,0	5,3		Oui	31,3	35,3	30,8	
Kalamu	23,2	5,9	25,4		Non	67,7	64,7	68,1	
Lukunga	31,9	38,2	31,1		Inconnu	1,0	0,0	1,1	
Ndjili	30,5	50,0	28,0		**Membres paralysés**				0.03*
Nsele	4,0	5,9	3,8		1	15,6	2,9	17,2	
**Statut vaccinal**				0,18*	≥ 2	84,4	97,1	82,8	
Inconnu	23,2	35,3	21,6						
< 4 doses	41,6	38, 2	42,05						
≥ 4 doses	35,2	26,5	36,4						

* Chi carré de Pearson

£ Test exact de Fisher.

Les principales manifestations cliniques relevées étaient une paralysie flasque aigue accompagné ou précédé de fièvre. La paralysie était asymétrique chez 3 patients sur 10. Les membres inférieurs étaient le plus fréquemment touchés (88,8% pour le membre inférieur droit et 88,1% pour le pour le membre inférieur gauche) que les membres supérieurs (15,6% pour le membre supérieur droit et 14,9% et pour le membre supérieur gauche). Il y avait significativement plus d'atteinte de plus d'un membre à la fois chez les cas de PFA du au PVS.

**Figure 1:**
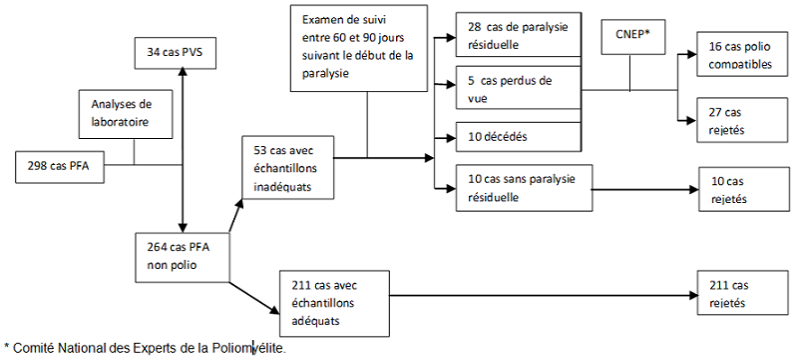
Classification des cas de PFA de décembre 2010 à décembre 2011

Les cas de PFA investigués étaient classifiés en cas de PVS et cas de PFA non poliomyélitiques, eux même catégorisés en cas poliomyélite compatibles, et cas « rejetés », c'est-à-dire définitivement classifiés comme cas de PFA non poliomyélitiques. La Figure 1 présente un diagramme de flux décrivant le processus d'investigation des cas de PFA notifiés de décembre 2010 à décembre 2011 et leur classification.

Les cas de PFA notifiés pendant cette période (298 cas) ont fait l'objet de prélèvement d’échantillons de selles qui a permis d'identifier 33 cas de PVS. Les 265 autres cas de PFA catégorisés « cas de PFA non poliomyélitique » ont été ensuite discriminés en « cas avec échantillons adéquats » (218 cas) ou « cas avec échantillons inadéquats » (53 cas). Les cas avec échantillons adéquats étaient directement « rejetés ». Après un suivi de 60 à 90 jours par l’équipe cadre de la zone de santé, les cas avec échantillons inadéquats étaient classés en 4 catégories : les cas sans paralysie résiduelle (10 cas) qui étaient « rejetés », les cas avec paralysie résiduelle (28 cas), les décédés (10 cas) et les perdus de vue (5 cas). Les trois dernières catégories (paralysies résiduelles, décédés et perdus de vue) ont fait l'objet d'analyses approfondies par le Comité National d'Experts de la Poliomyélite (CNEP). Les résultats de cette analyse permettaient de classifier certains cas comme « rejetés » (27 cas) et d'autres comme « cas poliomyélite compatibles » (16 cas). Les « cas poliomyélite compatibles » sont des cas qui auraient pu éventuellement être des cas de PVS si les échantillons de selles avaient été adéquats.

**Figure 2:**
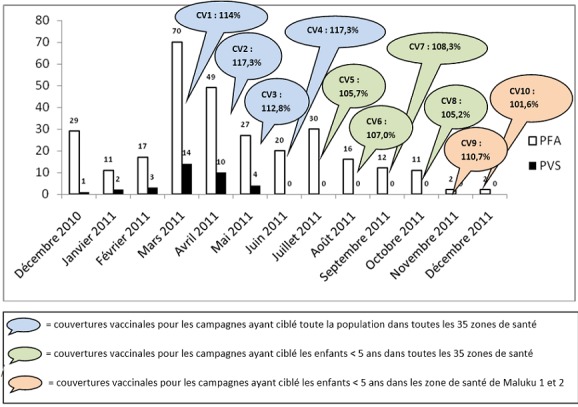
Evolution des cas de PFA et de PVS de décembre 2010 à décembre 2011 et campagnes de vaccination de riposte

En 2010, un cas de poliovirus avait été isolé dans 1 zone de santé et 33 cas en 2011 dans 14 zones de santé. La répartition des cas de PFA et de PVS notifiés mensuellement est présentée dans la Figure 2 ainsi que les différentes campagnes de riposte et les couvertures vaccinales réalisées par rapport à la population cible.

Le 1^er^ cas de PVS de type 1 est apparu au mois de décembre 2010 et le nombre de cas a augmenté progressivement pour atteindre le pic de 14 cas au mois de mars 2011. A partir de ce mois 4 campagnes de vaccination contre la poliomyélite ont été organisée mensuellement dans toute la ville ciblant toute la population avec des couvertures vaccinales variant de 113 à 117%. Ces campagnes ont été relayées par 4 autres qui avaient pour cible les enfants de 0 à 59 mois. Les couvertures vaccinales obtenues variaient de 105 à 118%. Après le 3^e^ passage de la campagne de vaccination ciblant toute la communauté, on a plus enregistré de nouveau cas de PVS. Le vaccin monovalent a été utilisé pour toutes les campagnes, sauf pour celle du mois de mais pour laquelle le vaccin bivalent 1 et 3 a été utilisé.

Pendant et après les campagnes de vaccination se déroulaient, un monitorage indépendant était organisé par l'OMS dans les aires de vaccination pour déterminer l'effectivité de la vaccination de toutes les personnes cibles et les éventuelles cas de non vaccination ainsi que leurs causes. La proportion des personnes non vaccinées dans les ménages a varié de 4 à 13% et en hors ménages de 7à 20% (Tableau 2).

**Tableau 2:** Données de monitorage indépendant au cours des différentes campagnes de vaccination ayant porté sur l'ensemble des zones de santé.

Mois	Nombre d'aires de vaccination	Nombre de personnes cibles vus	Personnes cible non vacciné dans les ménages	Personnes cible non vacciné hors ménages
	Effectifs	Effectifs	%	%
Mars	533	50352	4	12
Avril	639	41799	4	7
Mai	420	43331	8	10
Juin	480	55942	13	20
Juillet	544	23766	7	10
Août	525	23247	8	10
Septembre	525	24636	10	13
Octobre	558	24211	10	13

La performance des indicateurs de surveillance des cas de PFA étaient globalement bon pour la province de Kinshasa (Tableau 3). Néanmoins, on peut noter que seul 1 quart des zones de santé satisfaisaient au critère de performance de détection des cas de PFA retenu par la RDC en fonction des performances locales atteintes les années précédentes (≥ 5 cas pour 100 000 enfants 80 des cas investigués dans les 48h suivant la notification). 6 zones de santé sur 10 avait >80% des cas ayant fait l'objet de 2 prélèvement d’échantillons dans les 14 jours suivant le début de la paralysie. Et un peu plus de la moitié des zones de santé avaient une proportion de selles adéquates >80%.

**Tableau 3:** Performance des zones de santé dans la surveillance des cas de PFA (n=35)

Indicateur de performance	Zones de santé		Province
	n	%	%
**taux annualisé de PFA non poliomyélitique (cas pour 100 000 enfants <15 ans)**	35		7,8
≥ 5	9	25,7	
4,9 – 2	15	42,9	
< 2	11	31,4	
**Selles adéquates (%)**	35		82,9
≥ 80	18	51,4	
< 80	17	48,6	
**2 échantillons de selles prélevés dans les 14 jours (%)**	35		84,4
≥ 80	21	60,0	
< 80	14	40,0	
**PFA investigués dans les 48h suivant la notification (%)**	35		96,6
≥ 80	30	85,7	
< 80	5	14,3	

## Discussion

Après 6 années d'interruption de la circulation de PVS (2000 à 2005), la RDC est, depuis 2006 un pays à circulation rétablie qui connait des flambées épidémiques dans certaines de ses provinces. La dernière épidémie ayant sévi dans la capitale congolaise a connu 34 cas de PFA à PVS sur une période de 6 mois allant de décembre 2010 à mai 2011 avec un pic au mois de mars. Elle a concerné, en majorité, les sujets de 15 ans et plus avec un sexe ratio en défaveur du sexe masculin et n'a pas montré de lien significatif avec le statut vaccinal. Cette observation relative au statut vaccinal a néanmoins la faiblesse d’être basée principalement sur les déclarations, en particulier pour les sujets adultes (8). Une enquête sérologique menée en Italie a montré que la proportion des sujets séropositifs au PVS de type décroit avec l’âge. Une étude basée sur une évaluation sérologique du niveau d'immunité des sujets aurait été plus fiable [9, 10].

La proportion de sujets susceptibles relevés (41,6% de sujets avec moins de 4 doses de VPO), sans compter les sujets à statut vaccinal inconnu (23,1%), montrent que le terrain était favorable à l’éclosion d'une épidémie. Kidds et collaborateurs avaient effectivement montré, en Angola, que le risque de PFA à PVS était significativement moindre pour les sujets avec au moins 4 doses de vaccin antipolio [6].Les résultats obtenus par l'enquête MICS 2010 en RDC, qui indiquait une proportion d'enfants de 12 à 23 mois ayant reçu la 4e dose de vaccin antipolio (Polio3) du calendrier vaccinal de routine à seulement 68,3%, étayent cette observation d'un nombre important de sujet susceptibles dans la ville de Kinshasa [11].

Par ailleurs, la situation de proximité géographique et économique de Kinshasa par rapport au Congo Brazza et à l'Angola, ainsi que son rôle comme point de convergence administratif, politique et économique des provinces, dont certaines ont été frappées d’épidémie en 2010 (Bandundu et Kasaï occidental), peut avoir contribué à l’éclosion de l’épidémie de 2011 [4, 6, 7].

Le rôle des adultes non vaccinés comme agents de transmission a été évoqué notamment avec les épidémies du Congo Brazza et de la Namibie où le nombre de sujets adultes atteints était prédominant [8, 12]. En Angola, une étude cas-témoins a même relevé le rôle de la présence, dans les ménages des enfants atteints, d'adultes ayant fait un déplacement en dehors de la province [6]. Ces observations ont conduit certains chercheurs à suggérer des campagnes de vaccination périodiques ciblant les adultes [4]. A Kinshasa, outre les sujets de moins de 5 ans qui représentaient 20,6%, 70,7% des sujets touchés par la PFA à PVS avaient un âge compris entre 10 et 29 ans. Dans de contrainte de ressources limitées, des activités vaccinales périodiques ciblant les sujets de moins de 30 ans, à défaut de la population générale, pourraient contribuer à interrompre rapidement la chaîne de transmission.

La PFA accompagné de fièvre a dominé la symptomatologie avec une atteinte prédominante des membres inférieurs, souvent bilatérale.

Les zones de santé du district sanitaire de Ndjili et de Lukunga ont été les plus touchés. Dans ces deux districts sanitaires les zones concernées étaient toutes contigües, Kimbanseke, Kingasani, Biyela, Kikimi, Ndjili, Masina 1 pour le district sanitaire de Ndjili et Kintambo, Binza Météo, Selembao pour le district sanitaire de Lukunga, indiquant en réalité deux foyers caractérisés par une forte promiscuité et un environnement insalubre. Ceci rejoint les observations faites au Congo Brazza par Le Menach et collaborateurs ainsi que celles relevées pour l’épidémie de la Namibie en 2006 [4, 12, 13].

La riposte n'est intervenue que 3 mois après le déclenchement de l’épidémie, mais elle a été vigoureuse avec 4 passages ciblant toute la population qui a permis de juguler l’épidémie dès le 3e passage. Les campagnes se sont poursuivies chez les sujets de moins de 5 ans afin de réaliser au moins 4 passages après le dernier cas notifié selon les recommandations de l'OMS [13]. Les deux dernières organisées en novembre et décembre visaient à optimiser une zone tampon à forte couverture vaccinale entre le Bas Congo où sévissait encore une épidémie et la ville de Kinshasa.

Le retard dans la mise en ‘uvre de la riposte était dû principalement au manque de financement et l'attente de la mobilisation des ressources au niveau régional et international. En effet, le manque de financement constitue un grand risque pour le processus de l’éradication de la poliomyélite dans plusieurs pays [15].

Cependant, il faut prendre avec réserve les fortes couvertures > 100% affichées par les zones de santé. Elles peuvent traduire, certes, une difficulté de maîtrise du dénominateur, mais elles cachent probablement des sujets non atteints dont la proportion relevée au cours de la campagne par les moniteurs indépendant pouvait atteindre les 20%. Sur base des données issues de contrôles monitorages indépendant, les CDC estiment que le bilan de la vaccination en RDC est moyen en raison du fait, notamment que 5 des 11 provinces n'avaient pas réussi à ramener la proportion d'enfants non atteints à moins de 10% [16]. Les raisons pour les quelles ces enfants sont manqués étaient opérationnelles et sociales. Et parmi les raisons sociales, le taux de refus en début d'année 2011 était à 16%, avec des pics allant jusqu’à près 20% dans le Bas Congo et à près de 30% à Kinshasa, constituant le taux de refus le plus élevé au monde [17].

La discordance entre les taux de couverture vaccinale annoncés lors de la riposte (>100%) et le pourcentage relativement élevé d′enfant non vaccinés réellement observé par les moniteurs (Tableau 2) soulève le point crucial de la maîtrise de la population cible, à la fois sur le plan quantitatif (validité des recensements de population) et sur le plan de la répartition dans l′espace de la population et des mouvements de cette population. Le recensement scientifique de la population en préparation (la dernière remonte à 1984) et la mise en place d'un service fiable de gestion des flux de déplacements des populations dans les administrations communales et territoriales pourraient offrir une base fiable de planification et de suivi et évaluation des interventions en RDC [18].

Malgré un bilan de surveillance qualifié de globalement de médiocre pour le pays avec une proportion importante d’échantillons de selles inadéquats [16], Kinshasa présentait une surveillance performante et des investigations promptes. Les faiblesses notées en RDC étaient attribuées essentiellement à la partie Est du pays dont on connait les contraintes liées à la situation sécuritaire et à la faiblesse induite au niveau du système de santé [16].

A Kinshasa, les faiblesses relevées concernaient surtout les indicateurs tels que la proportion des selles adéquates et la proportion des selles prélevées dans les 14 jours. Ceci traduit essentiellement un retard dans la notification des cas. Il convient de relever que, le contexte épidémique avait permis le renforcement de la surveillance communautaire dans certaines zones de santé. Cela pourrait expliquer l'accroissement de la notification des cas de PFA dont le début de paralysie remontait à plus de 14 jours et tirer la performance des indicateurs vers le bas.

## Conclusion

Cet épisode de flambée épidémique interpelle tous les acteurs de la lutte contre la poliomyélite en RDC à redoubler d'effort dans la surveillance des PFA pour identifier à temps tout cas de PVS et riposter promptement de manière à progresser rapidement vers l’éradication. Les performances actuelles de la surveillance des PFA dans la ville de Kinshasa sont encourageantes. Mais il reste des efforts à fournir, notamment dans l'amélioration de la surveillance à base communautaire, afin d'amener l'ensemble des zones de santé au seuil de performance souhaité afin d'optimiser leurs capacités de détection des cas et leur réactivité pour une investigation prompte et de qualité. La prédominance des adultes parmi les cas notifiés traduit une susceptibilité importante dans cette tranche de la population généralement non concernée par les campagnes de vaccination. Des activités de vaccination supplémentaires ciblant cette catégorie de la population pourraient contribuer à interrompre plus rapidement la chaîne de transmission. En contexte de limitation des ressources, des campagnes ciblant la tranche d’âge de 0 à 30 ans pourraient couvrir plus de 90% des sujets les plus susceptibles.
